# Structural-Acoustic Coupling Effects on the Non-Vacuum Packaging Vibratory Cylinder Gyroscope

**DOI:** 10.3390/s131217176

**Published:** 2013-12-13

**Authors:** Xiang Xi, Xuezhong Wu, Yulie Wu, Yongmeng Zhang, Yi Tao, Yu Zheng, Dingbang Xiao

**Affiliations:** 1 College of Mechatronics Engineering and Automation, National University of Defense Technology, Changsha 410073, China; E-Mails: fordada@126.com (X.X.); xzwu@nudt.edu.cn (X.W.); ylwu_nudt@sina.com (Y.W.); zymnudt@163.com (Y.Z.); 2 Beijing Special Vehicle Institute, Beijing 100073, China; E-Mail: taotaoyiyi@163.com; 3 State key Laboratory of High performance Complex Manufacturing, Central South University, Changsha 410083, China; E-Mail: zhengyu@csu.edu.cn

**Keywords:** vibratory cylinder gyroscope, structural-acoustic coupling, resonant shell

## Abstract

The resonant shells of vibratory cylinder gyroscopes are commonly packaged in metallic caps. In order to lower the production cost, a portion of vibratory cylinder gyroscopes do not employ vacuum packaging. However, under non-vacuum packaging conditions there can be internal acoustic noise leading to considerable acoustic pressure which is exerted on the resonant shell. Based on the theory of the structural-acoustic coupling, the dynamical behavior of the resonant shell under acoustic pressure is presented in this paper. A finite element (FE) model is introduced to quantitatively analyze the effect of the structural-acoustic coupling. Several main factors, such as sealing cap sizes and degree of vacuum which directly affect the vibration of the resonant shell, are studied. The results indicate that the vibration amplitude and the operating frequency of the resonant shell will be changed when the effect of structural-acoustic coupling is taken into account. In addition, an experiment was set up to study the effect of structural-acoustic coupling on the sensitivity of the gyroscope. A 32.4 mV/°/s increase of the scale factor and a 6.2 Hz variation of the operating frequency were observed when the radial gap size between the resonant shell and the sealing cap was changed from 0.5 mm to 20 mm.

## 1. Introduction

The operation of vibratory cylinder gyroscopes relies on their symmetrical resonant shells which vibrate in a standing wave mode [1], therefore it is necessary to provide a relatively stable package for the vibration of the resonant shells. With the purpose of isolating any environmental disturbances, sealing caps are necessary components of these gyroscopes [2,3]. Disposition inside the sealing caps can be divided into two classes: vacuum treatment and non-vacuum treatment. For most Coriolis vibratory gyroscopes, such as micro silicon gyroscopes and hemispherical resonator gyroscopes, the vacuum treatment is usually employed to obtain a high quality factor (up to 10^6^) [4–6]. For a medium-sized metallic vibratory cylinder gyroscope, experimental studies show that its quality factor can increase 2–3 times (up to 20,000 – 30,000) in a vacuum [7], but a portion of these gyroscopes do not employ a vacuum treatment due to consideration of the production cost or because improvement of the quality factor is not deemed necessary [2,7,8]. In previous studies, the sealing caps of vibratory cylinder gyroscopes were used for sealing purposes only, and are not well known for any other effects.

It is considered that when the resonant shell vibrates at the operating frequency, there can be acoustic waves leading to considerable acoustic pressure inside the sealing cap [9]. The acoustic energy will affect the vibration of the resonant shell after reflecting back from the inner surface of the sealing cap. This phenomenon, known as structural-acoustic coupling, has received much attention in various fields. In theory, the acoustic behavior in an enclosed space has been well studied. Different boundary conditions, such as rectangular and cylindrical cavity shape or irregular enclosures all show significant effects on the distribution of the internal acoustic pressure [10–13]. Thus, the vibratory structure in an enclosed space is susceptible to variation of the boundary conditions. Experiments on structural-acoustic coupling were conducted as well by some researchers. It was reported that a finite thin cylindrical shell was excited by an internal acoustic source, and the pressure field of the shell could be measured using laser measurements [14]. Some other researchers investigated the structural-acoustic mode of the cylindrical duct, which was used for the geometrical shape design of a cavity device [15]. The effects of structural-acoustic coupling are of concern in the areas of vibration analysis, structural design and optimization, *etc.* [16]. In the field of Coriolis vibratory gyroscopes, studies on the structural-acoustic coupling effect are relatively scarce. Reference [17] shows that if the acoustic energy frequency components are close to the eigenfrequency of the mechanical structure in the MEMS gyroscope, undesirable motion of the sensor proof mass resulting in signal corruption can be produced. In [18,19] it is revealed that the performance of a vibratory cylinder gyroscope may also suffer from intense acoustic noises. However, little quantitative data or analysis has been presented. Most efforts to improve the gyroscope performance have concentrated on the vibratory structural elements, such as material processing, structural optimization and evolution. [2,4,7,20]. It is easily known that the vibration amplitude of the thin resonant shell is highly sensitive to the pressure exerted on its surface, and the resonant frequency of the resonant shell can be affected as well. Therefore, the performance of the vibratory cylinder gyroscopes will be uncertain when the structural-acoustic coupling effect is taken into account.

In this paper, the structural-acoustic coupling effect on a type of low cost vibratory cylinder gyroscope without vacuum packaging is studied. The dynamical behavior of the resonant shell is analyzed in theory. In addition, the coupling effects are quantitatively analyzed based on FE simulation. It is found that the operating frequency, the acoustic pressure and the vibration amplitude of the resonant shell are changed due to the gap variation between the resonant shell and the sealing cap. The degree of vacuum is also changed to observe the fluctuation of the shell vibration. Finally, experiments were implemented to study the mechanical sensitivity (scale factor) of the gyroscope which could be affected by the structural-acoustic coupling effect. The simulation and experimental results are useful for a deeper understanding of the performance of the vibratory cylinder gyroscopes without vacuum packaging.

## 2. Gyroscope Description

**Figure 1.**
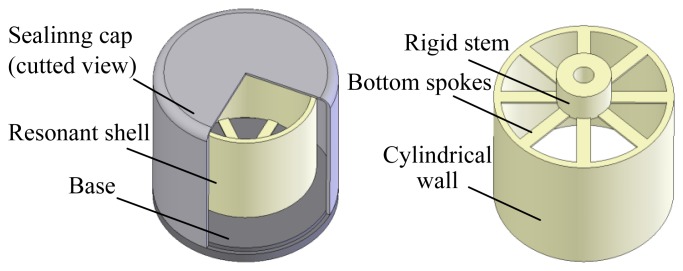
Physical structure of the vibratory cylinder gyroscope.

The typical vibratory cylinder gyroscope analyzed in this paper is shown in Figure 1. The physical part of the gyroscope is mainly made up by a resonant shell, a sealing cap and a base. The resonant shell is fabricated with nickel alloy such as Ni42CrTi and Ni-SPAN-C Alloy 902 [2]. Its structure comprises a rigid stem, eight bottom spokes and a cylindrical wall. The resonant shell is fixed on the base by a standard screw. The metallic sealing cap is used to package the resonant shell and isolate any environmental disturbances. The spoke structures on the bottom are designed to decrease the vibration coupling of the operating modes. Piezoelectric electrodes can be glued along the bottom spokes to excite the operating mode. It should be noted that the diameter of the resonant shell in this study is around 20 mm (medium-sized type), and the sealing cap is filled with a gas such as dry air. Despite operating under a non-vacuum condition, the gyroscope can provide tactical accuracy in a range of 1–5 °/h [2]. This characteristic makes the vibratory cylinder gyroscope competitive in terms of production cost.

The operating principle of the cylinder gyroscope is well understood. In brief, there is a pair of operating modes in the form of standing waves with a circumferential waves number of 2. The two modes referred to as the exciting mode and sensing mode are circumferentially spaced relative to each other by 45°. The exciting mode of the resonant shell is a standing wave vibration generated by piezoelectric or electrostatic excitation. When the gyroscope rotates, the sensing mode can be detected due to the Coriolis effect, hence the rotation rate of the gyroscope is obtained after circuit demodulation. For more information on the theory of the gyroscope, the reader can refer to the [21–23].

## 3. Theoretical Study of the Acoustic Coupling

Since the resonant shell of the vibratory cylinder gyroscope vibrates in the standing wave mode, its circumferential vibration is actually the topic in this study. Based on the elastic shell theory and the acoustic mode theory, a dynamic model is derived to qualitatively analyze the structural-acoustic coupling effect of the gyroscope.

**Figure 2.**
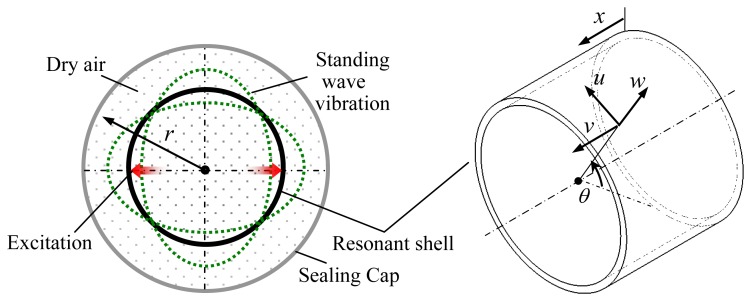
Schematic representation of the model.

The resonant shell has radius *R*_1_, thickness *h* and height *L*. The resonant shell is enclosed by a sealing cap of radius *R*_2_. Let *u*, *v* and *w* be, respectively, the tangential, axial and radial displacement of a point of the shell at the angular position *θ*, as shown in Figure 2. Let *ρ_r_* denote the mass density of the resonant shell, and it is assumed that the sealing cap is filled with dry air of density *ρ*_g_.

Before the acoustic effect is studied, the air damping should be taken into account. As a matter of experience, the amplitude of the shell vibration can be on the order of micrometers, and the operating frequency is below 20 kHz [2,20,24,25]. Therefore we can conclude that the vibration velocity of the shell is relatively low. Thus only the linear damping is introduced in the analysis. The quadratic or cubic damping can also be considered in the analysis, and similar results will be obtained [26,27]. The air resistance *f* is estimated by using the Navier–Stokes equation [28]:

(1) $$f<6\pi \mu_{g}\bar{R}\cdot \max\{\frac{dw}{dt}\}$$

where *μ_g_* is the viscosity coefficient of the air, *μ_g_* = 1.8 × 10^− 5^ N·s/m^−2^. *R̄* is the equivalent spherical radius which can be calculated by $\bar{R}=\sqrt[{3}]{\frac{3R_{1}^{2}L}{4}}$. The radial displacement *w* of the shell is given by:

(2) $$w=\frac{F_{ex}}{K}Qe^{\gamma x}\cos2\theta \cos\omega t$$

where *F_ex_* is the amplitude of the exciting force. *Q* is the quality factor of the shell, $Q=\frac{1}{2\xi }$(*ξ* is the damping coefficient). *K* is the bending stiffness which can be calculated by $K=\frac{9\pi EI}{2R^{3}}$(*E* is Young's modulus and *I* is the inertia moment of the shell), and ω is the eigenfrequency which can be calculated by $\omega =\frac{h}{R_{1}^{2}}\sqrt{\frac{3E}{5\rho_{r}(1-\mu^{2})}}$(*μ* is Poisson's ratio) [24]. *γ* is the axial wave number. Thus Equation (1) yields:

(3) $$\left|\frac{f}{F_{ex}}\right|<\frac{6\pi \omega \mu_{g}e^{\gamma L}\bar{R}Q}{K}$$

In order to estimate the influence of the air damping, several groups of geometric and material parameters are chosen by referring to the literature [2,20,24,25]. Assuming that 5 mm < *R*_1_ < 12.5 mm, 10 mm < *L* <30 mm, 0.5 mm < *h* <1.5 mm, 2,000 < *Q* < 30,000, 150 GPa < *E* < 250 GPa, the ratio between the air resistance *f* and the exciting force *F_ex_* can be obtained:

(4) $$\left|\frac{f}{F_{ex}}\right|<0.11$$

It can be seen that the air resistance is relatively low compared with the exciting force of the medium-size vibratory cylinder gyroscopes, therefore, the influence of the air damping is neglected in the following study.

Based on the theory of the elastic shell [24], the general solutions of the standing wave motion of the resonant shell are listed as follows:

(5) $$\begin{array}{c} v=V_{0}e^{\gamma x}\cos(2\theta )\cos(\omega t)\\ u=U_{0}e^{\gamma x}\sin(2\theta )\cos(\omega t)\\ w=W_{0}e^{\gamma x}\cos(2\theta )\cos(\omega t) \end{array}$$

where *V*_0_, *U*_0_ and *W*_0_ are constants which denote the amplitudes of the motion, and *γ* is the axial wave number.

The acoustic pressure will be generated inside the sealing cap. Note that the acoustic pressure comprises the component inside the resonant shell and the component between the resonant shell and the sealing cap. Let *p*_1_ and *p*_2_ denote the acoustic pressure exerting on the inner and outer surfaces of the resonant shell, respectively. For the acoustic wave inside the resonant shell (*r* < *R*_1_), the wave equation can be expressed as [15]:

(6) $$\frac{1}{r}\frac{\partial }{\partial r}(r\frac{\partial p_{1}}{\partial r})+\frac{1}{r^{2}}\frac{\partial^{2}p_{1}}{\partial \theta^{2}}+\frac{\partial^{2}p_{1}}{\partial x^{2}}-\frac{1}{c_{g}^{2}}\frac{\partial^{2}p_{1}}{\partial t^{2}}=-2\rho_{g}\frac{\partial^{2}w}{\partial t^{2}}\delta (\zeta -\zeta_{0})$$

where *δ*(*ξ − ξ*_0_) is the Dirac delta function in a coordinate normal to the inner surface of the resonant shell; *c_g_* is the acoustic velocity propagating in the air and *r* denotes the radial coordinate which is shown in Figure 2.

For the acoustic energy between the resonant shell and the sealing cap (*R*_1_ < *r* < *R*_2_), the acoustic pressure *p*_2_ can be obtained by using the acoustic wave equation for the closed space, which can be written as [29]:

(7) $$\frac{1}{r}\frac{\partial }{\partial r}(r\frac{\partial p_{2}}{\partial r})+\frac{1}{r^{2}}\frac{\partial^{2}p_{2}}{\partial \theta^{2}}+\frac{\partial^{2}p_{2}}{\partial x^{2}}-\frac{1}{c_{g}^{2}}\frac{\partial^{2}p_{2}}{\partial t^{2}}=2\rho_{g}\frac{\partial^{2}w}{\partial t^{2}}\delta (\zeta -\zeta_{0})-\rho_{g}\frac{\partial q}{\partial t}$$

where *q* represents the distribution of the volume velocity of the reflected acoustic wave. The intensity of the reflected acoustic wave is closely related to the acoustic source and the reflection boundary condition. The volume velocity distribution *q* can be expressed as [29]:

(8) q=|rp|e−α(R2−R1)∂w∂tδ(ζ'−ζ0')

where *α* is the acoustic attenuation coefficient, *r_p_* is the reflection coefficient determined by the properties of the materials on interface, and *δ*(*ξ^′^− ξ*_0_*^′^*) is the Dirac delta function in a coordinate normal to the inner surface of the sealing cap. The orientation of the incident acoustic wave is nearly perpendicular to the cap surface. Thus the reflection coefficient can be calculated by the following equation [29]:

(9) $$r_{p}=(1-\frac{\rho_{s}c_{s}}{\rho_{g}c_{g}})/(1+\frac{\rho_{s}c_{s}}{\rho_{g}c_{g}})e^{i\varphi \pi }$$

where *ρ_s_* is the mass density of the sealing cap, *c_s_* is the acoustic propagation velocity in the sealing cap, and *e^iφπ^* represents a phase variation due to the reflection.

It is known that the acoustic pressure can be expressed as a sum of the pressure distributions of the acoustic modes:

(10) $$\begin{array}{c} p_{1}(r,\theta ,x,t)=\overset{\infty }{\underset{n=0}{\text{∑}}}p_{1(n)}(t)\psi_{n}(r,\theta ,x)\begin{array}{cc} \begin{array}{c} , \end{array} & 0<r\leq R_{1} \end{array}\\ p_{2}(r,\theta ,x,t)=\overset{\infty }{\underset{n=0}{\text{∑}}}p_{2(n)}(t)\psi_{n}(r,\theta ,x)\begin{array}{cc} \begin{array}{c} , \end{array} & R_{1}\leq r\leq R_{2} \end{array} \end{array}$$

where *n* is the acoustic mode number. *p*_1(_*_n_*_)_(*t*) and *p*_2(_*_n_*_)_(*t*) are the amplitude functions of the acoustic modes, *ψ_n_* is the mode shape function. The mode shape function for any specific mode can be written as:

(11) $$\psi_{n}(r,\theta ,x)=J_{n}(k_{r}r)\cos(n\theta )\cos(k_{x}x)$$

where *J_n_*(*kr*) is the Bessel function of order *n*, and *k_x_* = *πR*_1_/*L*, $k_{r}^{2}+k_{x}^{2}=\frac{\omega^{2}}{c_{g}^{2}}$.

Taking the Helmholtz equation [15]:

(12) $$\nabla^{2}\psi_{n}+\frac{\omega_{n}^{2}}{c_{g}^{2}}\psi_{n}=0$$

where *ω_n_* is the nature frequency of the acoustic mode. It is given by [30]:

(13) $$\omega_{n}=c_{g}\sqrt{{\left(\frac{\alpha_{g}}{R_{1}}\right)}^{2}+{\left(\frac{k_{x}\pi }{L}\right)}^{2}}$$

where *α_g_* is the root of boundary characteristic equation for the resonant shell.

Then multiplying by *ψ_m_*, and integrating over the closed space, Equations (6) and (7) are represented as:

(14) $$\frac{d^{2}p_{1(n)}}{dt^{2}}+\omega_{n}^{2}p_{1(n)}=\frac{\rho_{g}c_{g}^{2}}{\Lambda_{1(n)}}\int_{S_{1}}\psi_{n}(R_{1},\theta ,x)\frac{\partial^{2}w}{\partial t^{2}}dS_{1}$$

(15) $$\frac{d^{2}p_{2(n)}}{dt^{2}}+\omega_{n}^{2}p_{2(n)}=-\frac{\rho_{g}c_{g}^{2}}{\Lambda_{2(n)}}\int_{S_{1}}\psi_{n}(R_{1},\theta ,x)\frac{\partial^{2}w}{\partial t^{2}}dS_{1}+\frac{\rho_{g}c_{g}^{2}\left|r_{p}\right|e^{-\alpha (R_{2}-R_{1})}}{\Lambda_{2(n)}}\int_{S_{2}}\psi_{n}(R_{2},\theta ,x)\frac{\partial^{2}w}{\partial t^{2}}dS_{2}$$

where *S*_1_ and *S*_2_ are, respectively, the surface areas of the resonant shell and the sealing cap. *V*_1_ and *V*_2_ denote the volume inside the resonant shell and the volume between the resonant shell and the sealing cap, respectively. $\Lambda_{1(n)}=\int_{V_{1}}\psi_{n}^{2}(r,\theta ,x)dV_{1}$, $\Lambda_{2(n)}=\int_{V_{2}}\psi_{n}^{2}(r,\theta ,x)dV_{2}$.

The vibratory cylinder gyroscope should operate in the standing wave mode. Thus it follows that:

(16) $$p_{1}(R_{1},\theta ,x,t)=P_{1}J_{2}(k_{r}R_{1})W_{p}\cos(2\theta )\cos(k_{x}x)e^{i\omega t}$$

(17) $$p_{2}(R_{1},\theta ,x,t)=[P_{21}J_{2}(k_{r}R_{1})+P_{22}J_{2}(k_{r}R_{2})]W_{p}\cos(2\theta )\cos(k_{x}x)e^{i\omega t}$$

where *P*_1_, *P*_21_ and *P*_22_ are coefficients obtained from Equations (14) and (15):

(18) $$P_{1}=-\frac{2k_{x}\pi \rho_{g}{c_{g}}^{2}\omega^{2}R_{1}[\gamma e^{\gamma L}\cos(k_{x}L)+k_{x}e^{\gamma L}\sin(k_{x}L)-\gamma ]}{(\omega_{n}^{2}-\omega^{2})(\gamma^{2}+k_{2}^{2})\sin(k_{x}L)\int_{0}^{R_{1}}rJ_{2}^{2}(k_{r}r)dr}$$

(19) $$P_{21}=\frac{2k_{x}\pi \rho_{g}{c_{g}}^{2}\omega^{2}R_{1}[\gamma e^{\gamma L}\cos(k_{x}L)+k_{x}e^{\gamma L}\sin(k_{x}L)-\gamma ]}{(\omega_{n}^{2}-\omega^{2})(\gamma^{2}+k_{2}^{2})\sin(k_{x}L)\int_{R_{1}}^{R_{2}}rJ_{2}^{2}(k_{r}r)dr}$$

(20) $$P_{22}=-\frac{2k_{x}\pi \left|r_{p}\right|e^{-\alpha (R_{2}-R_{1})}\rho_{g}{c_{g}}^{2}\omega^{2}R_{2}[\gamma e^{\gamma L}\cos(k_{x}L)+k_{x}e^{\gamma L}\sin(k_{x}L)-\gamma ]}{(\omega_{n}^{2}-\omega^{2})(\gamma^{2}+k_{2}^{2})\sin(k_{x}L)\int_{R_{1}}^{R_{2}}rJ_{2}^{2}(k_{r}r)dr}$$

The damping force due to the acoustic pressure can be calculated by:

(21) $$F_{ac}=2\int_{0}^{L}\int_{-\pi /4}^{\pi /4}(p_{1}+p_{2})R_{1}d\theta dx$$

Therefore the radial vibration of the resonant shell including the consideration of acoustic pressure can be expressed as:

(22) $$w=\frac{F_{ex}-F_{ac}}{K}Qe^{\gamma x}\cos2\theta \cos\omega t$$

Substituting the general solution Equation (5) into Equation (22) yields a final form of the radial vibration:

(23) $$w=\frac{F_{ex}Q}{K-QM}e^{\gamma x}\cos(2\theta )\cos(\omega t)$$

where $M=\frac{2R_{1}}{k_{x}}[P_{1}J_{2}(kR_{1})+P_{21}J_{2}(kR_{1})+P_{22}J_{2}(kR_{2})]$.

**Figure 3.**
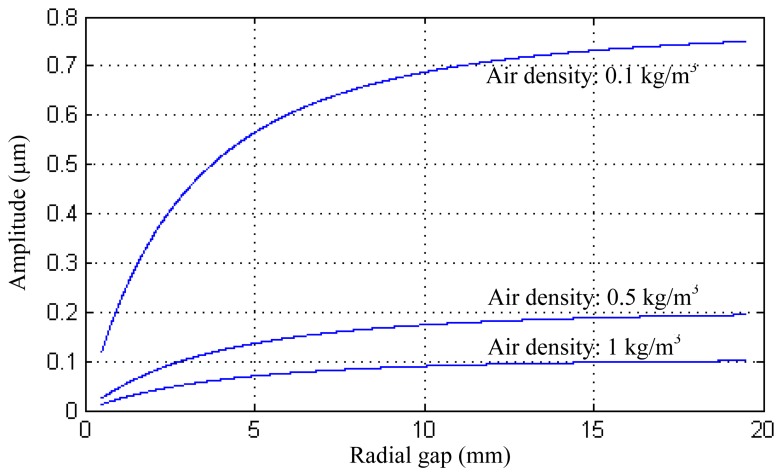
The amplitude including the consideration of structure-acoustic coupling effect.

Equation (23) reveals the effect of acoustic energy on the amplitude of the shell. The influence factors include the sizes of the structure and the properties of the acoustic propagation medium. A numerical example is also given to illustrate the theoretical study. In the example, the excitation forces *F_ex_* = 10^−3^ N; the resonant shell radius *R*_1_ = 12.5 mm, and its mass density *ρ_r_* = 7,780 kg/m^3^, Young's modulus *E* = 210 GPa, material damping *ξ* = 10^−5^, and the sealing cap density *ρ_s_* = 2,690 kg/m^3^. The acoustic velocity *c_g_* can be obtained from an acoustic handbook [22]. The radial displacement of the resonant shell under different radial gap and air density is shown in Figure 3. It turns out that a large radial gap and a low air density can increase the amplitude of the shell vibration. However it should be noted that the numerical results in this section are mainly based on a basic shell model which may result in a loss of accuracy. The actual structure of a vibratory cylinder gyroscope is more complicated. For example, the bottom spokes of the resonant shell make the acoustic boundary conditions extremely difficult, but the basic shell model is helpful for understanding the variation trend of the vibration parameter, and the mathematical model is more time-saving than a FE model which needs to solve a large number of equations.

It has been known that when the vibratory cylinder gyroscope rotates at an angular velocity Ω, the sensing mode of the resonant shell begins to appear, and it is oriented at 45° relative to the exciting mode. The amplitude of the generated standing wave is proportional to the angular velocity and the amplitude of exciting vibration. Thus the angular velocity of the gyroscope is obtained after electronical processing. Since the mechanical sensitivity of the gyroscope is proportional to the amplitude of the shell vibration, the scale factor of the gyroscope will be changed due to the structural-acoustic coupling effect.

## 4. Simulation Analysis

### 4.1. FE Modeling

In order to improve the analytical accuracy of the model, the FEM software ANSYS is employed to analyze the acoustic coupling effect of the vibratory cylinder gyroscope. A group of typical geometry parameters are used to build the initial model, as presented in Table 1 [31]. The material of the metallic resonant shell possesses isotropic and homogeneous properties, which are listed in Table 2. The sealing cap is made of aluminum, and its properties are also given in Table 2. The gas filled in the sealing cap is homogeneous dry air. The element types used to build the structure model (including the resonant shell and the sealing cap) and the fluid model are, respectively, SOLID95 and FLUID30. The length of the element is about 0.5 mm, and the number of the elements is 49,799 after meshing. The FE method is also available for the vibratory cylinder gyroscopes of other sizes.

The excitation methods of the vibratory cylinder gyroscope can be various, such as magnetic excitation, piezoelectric excitation and electrostatical excitation. The excitation position can be located at the bottom disk or on the cylindrical wall [2,18]. In this study, a pair of uniform exciting forces of 10^−3^ N is applied on the bottom of the resonant shell, which is marked in Figure 3. Under this exciting force, the amplitude of the standing wave vibration of the resonant shell is close to the experimental value (micrometer-order) [32]. The main steps for the structural-acoustic coupling analysis are as follows:

(1) First, modal analysis is performed to obtain the eigenfrequency of the resonant shell.
(2) Then, structural-acoustic coupling analysis is implemented by using the harmonic excitation. It is considered that there would be a small variation of the resonant frequency of the resonant shell when the acoustic effect is taken into account. Thus the harmonic analysis can provide more accurate solutions in this study. The range of the sweep-frequency is ±10 Hz around the eigenfrequency obtained from the modal analysis. The number of the substeps is set to 100, which is sufficient enough to get a convergence solution. Thus the accurate resonant frequency of the resonant shell can be obtained.

**Figure 4.**
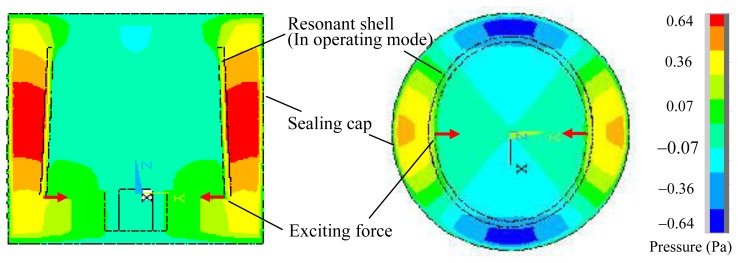
Acoustic pressure contour of the vibratory cylinder gyroscope.

By using the above method, the acoustic pressure contour of the vibratory cylinder gyroscope at the resonant frequency is obtained, as shown in Figure 4. For the gyroscope with the parameters listed in Table 1 and 2, the resonant frequency of the resonant shell is 4,423.2 Hz, the maximum acoustic pressure is 0.64 Pa, and the amplitude of the shell vibration is 0.36 μm. Since the resonant shell is in its standing wave mode, an elliptical deformation of the resonant shell can be observed in Figure 4. It can be seen that the distribution of the acoustic pressure is closely related to the vibration of the resonant shell. In the radial direction, the acoustic pressure is symmetrical, and reaches its maximum value at the antinodes of the vibrating shell. In the axial direction, a gradient variation of the acoustic pressure can be found, and the acoustic pressure between the resonant shell and the sealing cap is much larger than that inside the resonant shell. Due to the clearance on the open end of the resonant shell, small scattering of the acoustic pressure is also observed.

### 4.2. Analysis

#### 4.2.1. Consideration of the Radial Gap Size

**Figure 5.**
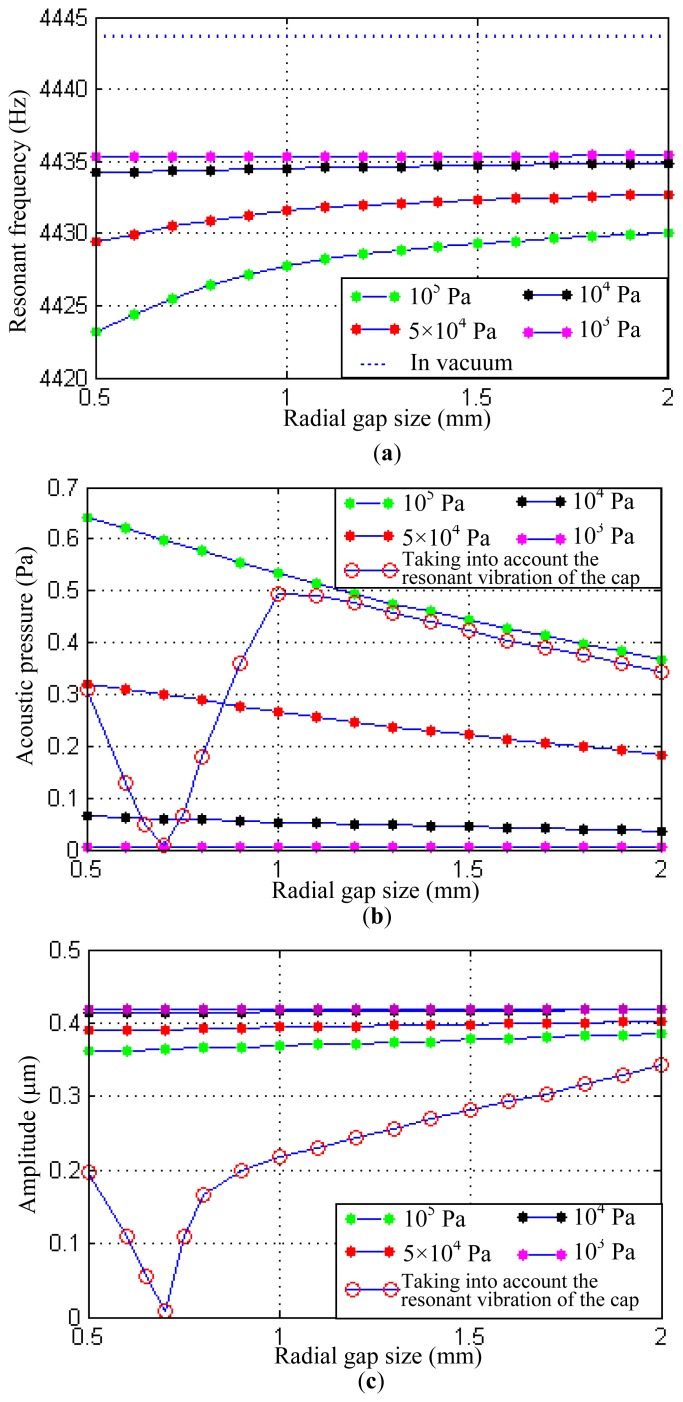
Acoustic effect under different radial gaps and vacuum degree. (**a**) Variation of the resonant frequency. (**b**) Variation of the acoustic pressure. (**c**) Variation of the amplitude.

Based on the theoretical analysis in Section 3, several main factors affecting the vibration of the resonant shell are analyzed in this part. Firstly, keeping the parameters of the resonant shell consistent with those in Table 1 and changing the size of the sealing cap, the structural-acoustic coupling under the varying radial gaps can be obtained. In the simulation, the gap size between the resonant shell and the sealing cap ranges from 0.5 mm to 2 mm. Larger gap sizes are not included since that will sharply increase the computing time, and the chosen gap range in this study is representative to illustrate the structural-acoustic coupling effect. The simulation results are shown in Figure 5. They indicate that the resonant frequency of the resonant shell increases as the widening of the gap size. Under normal atmospheric pressure (10^5^ Pa), the variation of the resonant frequency is 6.8 Hz when the gap size is extended from 0.5 mm to 2 mm. However the increase rate of the resonant frequency gradually falls as the widening of the gap size, as shown in Figure 5a.

It can be found that the variation of the resonant frequency is affected by the acoustic pressure exerting on the shell surface. Figure 5b shows the change of the maximal acoustic pressure in the sealing cap. The variation of the acoustic pressure is 0.274 Pa when the gap size is extended from 0.5 mm to 2 mm (under 10^5^ Pa atmospheres), and it can be seen that a relatively high acoustic pressure will decrease the resonant frequency of the resonant shell.

A large acoustic pressure can also limit the amplitude of the shell vibration. The acoustic pressure decreases as the gap size extends from 0.5 mm to 2 mm. However the amplitude of the shell vibration will be increased from 0.361 μm to 0.385 μm, as shown in Figure 5c. The tendency of the amplitude variation is similar to that revealed in the theoretic analysis.

It is noted that when the eigenfrequency of the sealing cap is close to that of the resonant shell, the eigenmode of the sealing cap may be excited due to the periodical acoustic pressure. As a particular example, a sealing cap with radius 13 mm, height 20 mm and thickness 1.25 mm has an eigenfrequency of 4,577 Hz, which is close to the operating eigenfrequency of the resonant shell (about 4,400 Hz). In this case, the vibration energy of the resonant shell will transfer to the sealing cap. Thus the amplitude of the shell vibration can be largely decreased. In Figure 5b,c, V-shape curves can be found when the resonant vibration of the sealing cap is taken into account. The amplitude of the resonant shell decreases and reaches the lowest value at the position of 0.7 mm gap size. Then the amplitude increases from 0.05 μm to 0.34 μm when the gap size is extended to 2 mm.

The study of the resonance phenomenon of the sealing cap would be helpful for the seal design of the vibratory cylinder gyroscope. The radial gap size can also be changed by decreasing the size of the resonant shell, and similar results will be obtained.

#### 4.2.2. Consideration of the Degree of Vacuum

In most cases, vacuum treatment is used to eliminate the damping of the air and increase the quality factor of the resonant structures. However, different degrees of vacuum can also affect the structural-acoustic coupling effect of the vibratory shell. In this section, the degree of vacuum is changed to obtain the resonant frequency, the acoustic pressure and the amplitude of the shell vibration.

The degree of vacuum *P* can be calculated from the ideal gas equation:

(27) $$P=\rho_{g}R_{g}T/M$$

where *ρ_g_* is the mass density of the air, *R_g_* is the universal gas constant, *T* is the temperature, *M* is the molar mass of the air. Therefore the degree of vacuum can be set by changing the air density.

Several different degrees of vacuum are considered in this study: 10^3^, 10^4^, 5 × 10^4^ and 10^5^ Pa, respectively. The simulation results are also shown in Figure 5. It indicates that a low degree of vacuum can significantly reduce the structural-acoustic coupling effect. When the degree of vacuum is 10^3^ Pa, the variations of the resonant frequency, the acoustic pressure and the amplitude are only 0.1 Hz, 0.00274 Pa and 0.0003 μm, respectively. Therefore a degree of vacuum of 10^3^ Pa is low enough to eliminate the structural-acoustic coupling effect for the vibratory cylinder gyroscopes studied in this paper. It should be noted that a lower degree of vacuum may have further influence on the resonant shell such as increase of the quality factor, but that is not the focus of this study.

The influence of the structural-acoustic coupling effect on the gyroscope also depends on the parameters such as material damping and stiffness coefficient. The acoustic reflection coefficient may affect the acoustic pressure as well. However, these parameters vary little for the metallic materials which are commonly used to fabricate the resonant shell and the sealing cap, therefore they are not studied in this paper.

## 5. Influence on Scale Factor of the Gyroscope

### 5.1. Experimental Setup

It is well known that for the Coriolis vibratory gyroscope, its mechanical sensitivity is proportional to the amplitude of the proof mass. The sensitivity *S* of such a vibratory gyroscope is determined by [33]:

(28) $$S=\frac{2\omega_{x}X}{\omega_{y}^{2}{\left[{\left(1-r_{\omega }^{2}\right)}^{2}+{\left(r_{\omega }/Q\right)}^{2}\right]}^{1/2}}$$

where *X* is the amplitude of the driving motion, *ω_x_* and *ω_y_* are, respectively, the resonant frequencies of the driving and sensing modes, and *Q* is the mechanical quality factor of the sensing motion, *r_ω_* = *ω_x_*/*ω_y_*. Thus the sensitivity of the gyroscope can be influenced by the amplitude variation caused by the structural-acoustic coupling effect. Scale factor is usually employed for sensitivity calculation, which represents the ratio between the change in gyroscope output and the relevant angular velocity variation [34].

Previous study reveals that the vibration of the resonant shell is mainly affected by the combined factors of the radial gap size and the degree of vacuum. Considering that the gyroscopes studied in this paper is a type of low cost cylinder gyroscopes without vacuum treatment, the major factor affecting the sensitivity of the gyroscope is therefore the size of the sealing cap. An experiment was implemented to study the sensitivity of the gyroscope by changing the size of the sealing cap. Since the degree of vacuum was a constant value during the experiment, the mechanical quality factor of the resonant shell would be accordingly unchanged. Thus the mechanical sensitivity of the gyroscope was mainly influenced by the acoustic energy inside the sealing cap.

**Figure 6.**
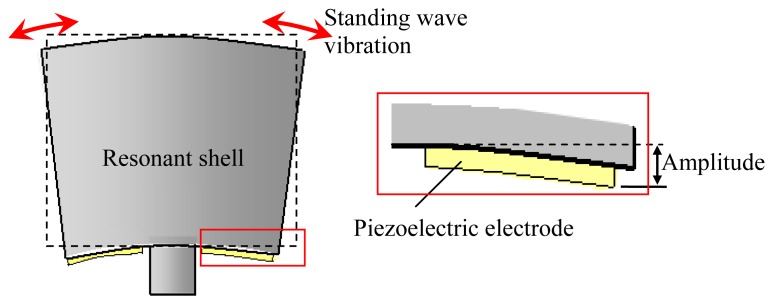
Signal output via piezoelectric electrodes.

In the study, the amplitude of the shell vibration is converted to the output voltage signal by using the sensing piezoelectric electrodes attached on the bottom of the resonant shell, as shown in Figure 6.

The experiment included the following steps: first, a control circuit was designed to obtain the output signal of the gyroscope. Then the gyroscope was fixed on a turntable for the sensitivity test. Finally, a series of scale factors were obtained by changing the radial gap size between the resonant shell and the sealing cap. The experiment was conducted in a thermostatic and noiseless room (20 °C).

**Figure 7.**
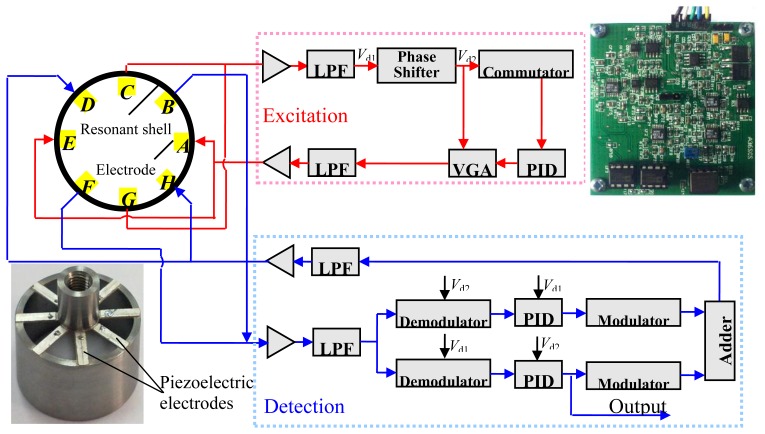
The block diagram of the control method and a photo of the control circuit.

Figure 7 shows the block diagram and the photo of the control circuit which includes an excitation control loop and a detection control loop. The excitation control loop excites the exciting mode of the resonant shell and keeps the exciting voltage at a constant value. Thus the gyroscope can operate in the exciting mode by the self-excited oscillation circuit. A *π*/2 shifter is designed to satisfy the self-excited oscillation condition that phase delay of the excitation loop is 2*nπ* (*n* is the nature number). Piezoelectric electrodes *A* and *E* receive a sinusoidal exciting voltage, and piezoelectric electrodes *C* and *G* output voltage proportional to the amplitude of the vibration to voltage gain amplifier, which provides negative feedback control to stabilize the exciting force at a constant value. The force-balance loop compensates for the Coriolis force induced by the input angular velocity and restrains the sensing mode of the resonant shell. When the resonator rotates about its central axis, the sensing mode of the resonator is generated due to the Coriolis effect, and the piezoelectric elements *B* and *F* output the sensing signal. This signal is demodulated and modulated with signals from excitation loop to form a compensation signal. The compensation signal is applied to the piezoelectric electrodes *D* and *H*, which generates compensation forces to balance the Coriolis force and restrain the sensing mode. Therefore, the resonant shell keeps on operating in the exciting mode. The feedback signal from the output of the PID regulator is proportional to the input angular velocity. Thus the angular velocity signal of the gyroscope could be obtained.

### 5.2. Results and Discussion

**Figure 8.**
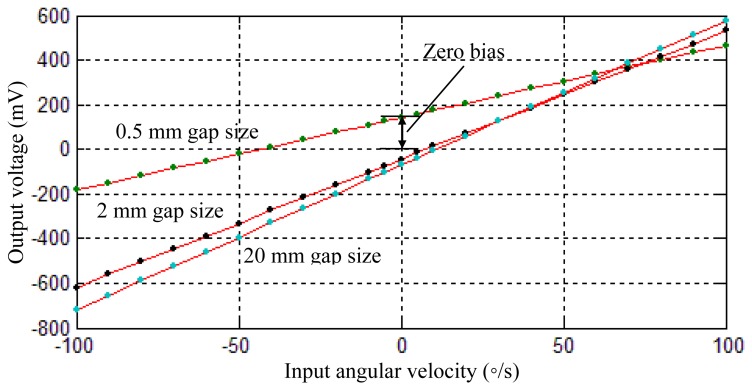
Scale factor test under different radial gap sizes.

Figure 8 shows the sensing voltages of the gyroscope under different input angular velocities. The scale factor was calculated accordingly. It can be found that the gyroscope with 0.5 mm radial gap size has the lowest scale factor of 32.5 mV/°/s. As the radial gap size is increased to 2 mm, the scale factor is promoted to 57.5 mV/°/s, which is 76.9% larger than the lowest value. However, for the radial gap of 20 mm, the further improvement of the scale factor is not significant. Its scale factor is 64.9 mV/°/s, which is only 10.3% larger than that of a 2 mm gap size.

The resonant frequency of the resonant shell is changed as well in the experiments. For the 0.5 mm radial gap size, the resonant frequency of the resonant shell is 3,963.8 Hz; for the 2 mm radial gap size, the resonant frequency is increased to 3,969.3 Hz; and for the 20 mm radial gap size, the resonant frequency is 3,970 Hz. These experiment results show a trend similar to those obtained from the previous simulation. It is noted that the zero bias of the gyroscope is also different for the different sealing caps, as shown in Figure 8. This phenomenon may be due to the small mode deflection caused by different sealing conditions, which made the voltage output at the antinodes of the resonant shell not exactly zero.

It can be concluded that for the vibratory cylinder gyroscope without vacuum packaging, the scale factor is affected by the radius of the sealing cap, that is, the structural-acoustic coupling has an effect on the gyroscope's scale factor. Due to this effect, a narrow radial gap between the resonant shell and the sealing cap will limit the amplitude of the shell vibration. Large radial gaps tend to promote the scale factor of the gyroscope since the vibration amplitude is increased. Considering that a large sealing cap will increase the whole volume of the gyroscope as well, the cap size should be designed to an appropriate value by using suitable theoretical, simulation or experimental methods. Though the scale factor of the gyroscope can be amplified by regulating the parameters of the control circuit, this will increase the electric noise of the circuit accordingly.

## 6. Conclusions

In this paper, the acoustic coupling effect on the vibratory cylinder gyroscopes is studied. Theoretical and simulation results show that the amplitude of the shell vibration is related to the acoustic pressure in the sealing cap. Widening the gap size between the resonant shell and the sealing cap or lowering the degree of vacuum can increase the amplitude of the shell vibration, therefore, the mechanical sensitivity of the vibratory cylinder gyroscope can be partly improved using these methods.

## Figures and Tables

**Table 1.** Geometric parameters of the gyroscope.

**Parameter**	**Value**

Height and thickness of the cylindrical wall	15 mm and 1 mm
Radius of the cylindrical wall	12.5 mm
Thickness of the bottom disk	0.3 mm
Height and radius of the sealing cap	20 mm and 13 mm

**Table 2.** Material properties of the gyroscope and the dry air.

**Parameter**	**Value**
Young's modulus of the resonant shell	210 GPa
Poisson's ratio of the resonant shell	0.28
Material damping of the resonant shell	1 × 10^−5^
Mass density of the resonant shell	7,780 kg/m^3^
Young's modulus of the sealing cap	62 GPa
Poisson's ratio of the sealing cap	0.3
Mass density of the sealing cap	2,690 kg/m^3^
Mass density of the dry air	1.2 kg/m^3^
Acoustic velocity in the dry air	340 m/s
